# Integration of microRNA and mRNA expression profiles in the skin of systemic sclerosis patients

**DOI:** 10.1038/srep42899

**Published:** 2017-02-17

**Authors:** Bin Zhou, Xiao Xia Zuo, Yi Sha Li, Si Ming Gao, Xiao Dan Dai, Hong Lin Zhu, Hui Luo

**Affiliations:** 1 Department of Rheumatology, Xiangya Hospital, Central South University, 87 Xiangya Road, Changsha, Hunan 410008, People’s Republic of China

## Abstract

MicroRNAs (miRNAs) play important roles in the fibrosis of systemic sclerosis (SSc). However, the underlying miRNA-mRNA regulatory network is not fully understood. A systemic investigation of the role of miRNAs would be very valuable for increasing our knowledge of the pathogenesis of SSc. Here, we combined miRNA and mRNA expression profiles and bioinformatics analyses and then performed validation experiments. we identified 21 miRNAs and 2698 mRNAs that were differentially expressed in SSc. Among these, 17 miRNAs and their 33 target mRNAs (55 miRNA-mRNA pairs) were involved in Toll-like receptor, transforming growth factor β and Wnt signalling pathways. Validation experiments revealed that miR-146b, miR-130b, miR-21, miR-31 and miR-34a levels were higher whereas miR-145 levels were lower in SSc skin tissues and fibroblasts, normal fibroblasts and endothelial cells that were stimulated with SSc serum. ACVR2B, FZD2, FZD5 and SOX2 levels were increased in SSc skin fibroblasts, normal fibroblasts and endothelial cells that were stimulated with SSc serum. We did not identify any negative correlations among these miRNA-mRNA pairs. miR-21 was specifically expressed at higher levels in SSc serum. Six miRNAs and 4 mRNAs appear to play important roles in the pathogenesis of SSc are worth investigating in future functional studies.

Systemic sclerosis (SSc) is a complex heterogeneous autoimmune disease that is characterized by inflammation, vasculopathy, and extensive fibrosis^1^,^2^. Basis on the extent of skin involvement, patients are categorized as having either limited cutaneous systemic sclerosis (lSSc) or diffuse cutaneous systemic sclerosis (dSSc)^3^. The pathogenesis of SSc is dominated by vascular changes. Vascular injury and endothelial activation induce fibroblast activation and subsequent fibrosis, which leads to an uncontrolled inflammatory reaction that results in irreversible scarring and eventual organ failure. The transforming growth factor-β (TGF-β). canonical Wnt, and Toll-like receptor (TLR) signalling pathways are the best studied pathways which play important roles in driving collagen production and promoting fibrotic matrix deposition^4^.

The exact cause of SSc currently remains elusive but is likely to involve the effects of environmental factors on genetically primed individuals^5^,^6^. Epigenetic factors, such as microRNAs, DNA methylation, histone modification and long non-coding RNA, have been widely studied as potential contributors to the diversity of clinical symptoms and laboratory findings that have been documented in SSc patients^7^,^8^,^9^.

miRNAs are non-coding RNAs that are ~22 nucleotides in length and function as intracellular regulators of gene expression. miRNAs play key biological roles by modulating both gene and protein levels by destabilizing transcripts and inhibiting protein translation, respectively^10^,^11^. Many miRNAs (e.g., miR-21^12^. miR-29^13^, and miR130b^14^) have been shown to be aberrantly expressed in SSc patients and therefore potential contributors to its pathogenesis. A single miRNA can target many genes, multiple miRNAs can regulate a single gene^15^, and miRNAs can be regulated by targeted interactions^16^. Hence, miRNAome and mRNAome interactions form a complicated network. However, most studies have focused on identifying the functions of single miRNAs mainly using *in vitro* experiments, such as transfection or luciferase activity assays, which may not reflect their real *in vivo* effects^17^. It would therefore be of substantial value to identify the targets of miRNAs to shed light on their complex regulatory networks. Systematic analyses and the integration of miRNAs and transcriptomics are approaches that may provide new insights into the pathogenesis of SSc in addition to important biomarkers and therapeutic targets^18^.

In our previous miRNA array experiments, we found aberrantly expressed miRNAs in dSSc and lSSc lesioned skin and 21 miRNAs were altered in both types of tissues^19^. We hypothesized that these 21 miRNAs might play fundamental roles and regulate important pathways in SSc. In the current study, we integrated these 21 miRNAs and whole mRNA expression profiles to analyze the functions of miRNAs at the genome level. First, we used a TargetScan database and IPA to select all of the predicted mRNA targets of the 21 miRNAs. This analysis was then enriched by a further bioinformatic analysis. We selected the predicted mRNAs that were involved in important biological pathways (e.g., the TLR, TGF-β and Wnt signalling pathways) in SSc. Next, we analyzed the gene expression profiles of these markers in SSc skin tissues (NCBI GEO Database, GSE9285) and identified the genes that were differentially expressed in SSc. Third, we combined these predicted mRNAs with the differentially expressed genes. Finally, we validated these findings regarding differentially expressed miRNAs and mRNAs using SSc skin tissues, SSc skin fibroblasts, normal fibroblasts or endothelial cells that were stimulated with SSc serum.

## Results

### Differentially expressed miRNAs in the SSc skin tissues

In our previous study, we used a custom microarray platform to evaluate the miRNA expression profiles of skin tissues obtained from SSc patients. This microarray set included nine biologically independent samples, including three normal skin samples, four dSSc skin samples, and two lSSc skin samples. Each skin sample was derived from a single specimen. We identified a total of 21 miRNAs that were similarly altered in both dSSc and lSSc (13 miRNAs were upregulated and eight miRNAs were downregulated)^19^.

**Figure 1:**
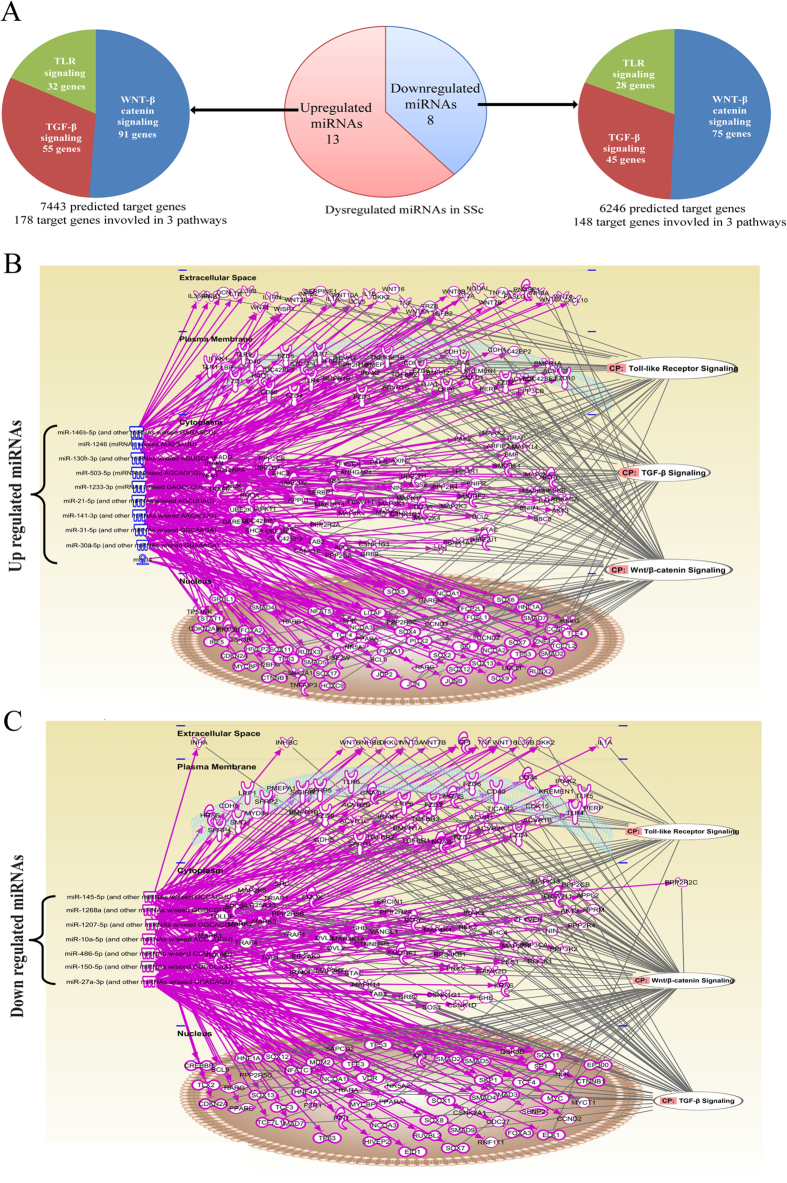
Dysregulated miRNAs and their target genes in SSc skin tissues. (**A**) Dysregulated miRNAs in SSc and their predicted target genes and pathways. (**B**) Ten upregulated genes and their predicted target genes were found to be involved in the TLR, TGF-β and Wnt signalling pathways. (**C**) Seven downregulated genes and their predicted target genes were found to be involved in the TLR, TGF-β and Wnt signalling pathways.

We identified 13689 predicted conserved target mRNAs for the 21 previously identified miRNAs using the TargetScan database. Among these, 326 of the target mRNAs (2.38%) were involved in the TLR, TGF-β and Wnt signalling pathways, including 178 mRNAs that might be targeted by 10 upregulated miRNAs (hsa-miR-146b-5p, hsa-miR-503, hsa-miR-1246, hsa-miR-1233, hsa-miR-130b, hsa-miR-141, hsa-miR-21, hsa-miR-30a, hsa-miR-31 and hsa-miR-34a) and 148 mRNAs that might be targeted by 7 downregulated miRNAs (hsa-miR-10a, hsa-miR-1207-5p, hsa-miR-1268, hsa-miR-150, hsa-miR-27a, hsa-miR-486-5p and hsa-miR-145) (Fig. 1A,B and C). All of these data are shown in Supplemental Table 1.

### mRNAs were differentially expressed in SSc skin tissues

**Figure 2:**
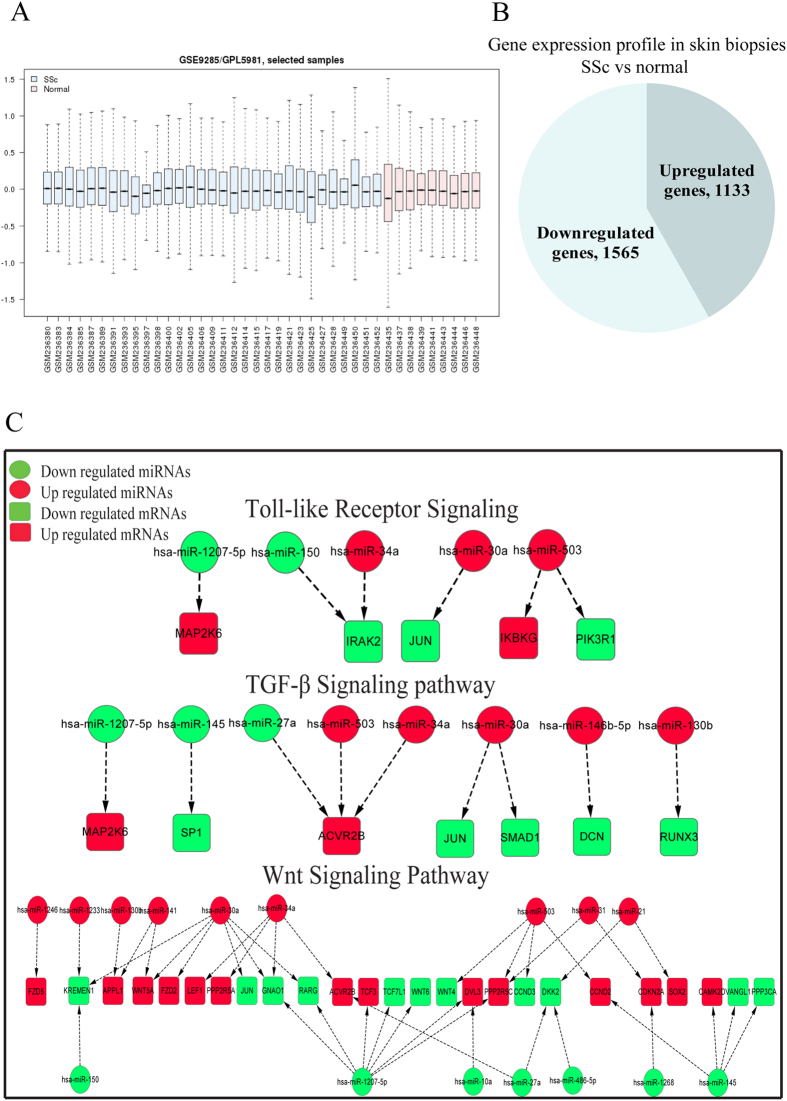
Differentially expressed genes in SSc skin tissues. (**A**) Microarray data for 31 SSc and 9 normal control samples were obtained from the NCBI GEO Database (GSE9285). (**B**) In all, 2698 genes were dysregulated in SSc skin tissues (1133 were upregulated, and 1565 were downregulated). (**C**) Networks representing the TLR, TGF-β and Wnt pathways were manually curated by combining KEGG pathway information with data for the miRNAs and their predicted targets. Nodes represent dysregulated miRNAs (circle) and genes (square) in the pathways. Node colours (red for upregulation and green for downregulation) represent fold changes in the corresponding miRNAs or genes.

We analysed the gene expression data using the Gene Expression Omnibus (http://www.ncbi.nlm.nih.gov/geo/GSE accession number GSE9285), which contains 31 SSc skin tissues and 9 normal skin tissues that were obtained from forearm skin biopsies^20^. A total of 2698 genes (including 1133 upregulated and 1565 downregulated genes) were found to be differentially expressed between SSc and normal skin tissues (31 SSc vs 9 normal control samples with fold changes ≥1.2, P < 0.05; Fig. 2A and B). We combined these differentially expressed genes with the previously obtained predicted miRNA target genes and matched 33 gene pairs that resulted in 55 miRNA-mRNA pairs. In total, 5 miRNAs and 5 mRNAs were dysregulated in the TLR signalling pathway, 8 miRNAs and 7 mRNAs were dysregulated in the TGF-β signalling pathway, and 16 miRNAs and 25 mRNAs were dysregulated in the Wnt signalling pathway (Fig. 2C). All data are shown in Supplemental Table 2.

### Validation of miRNAs and mRNAs that were differentially expressed in SSc skin tissues and primary skin fibroblasts

**Figure 3:**
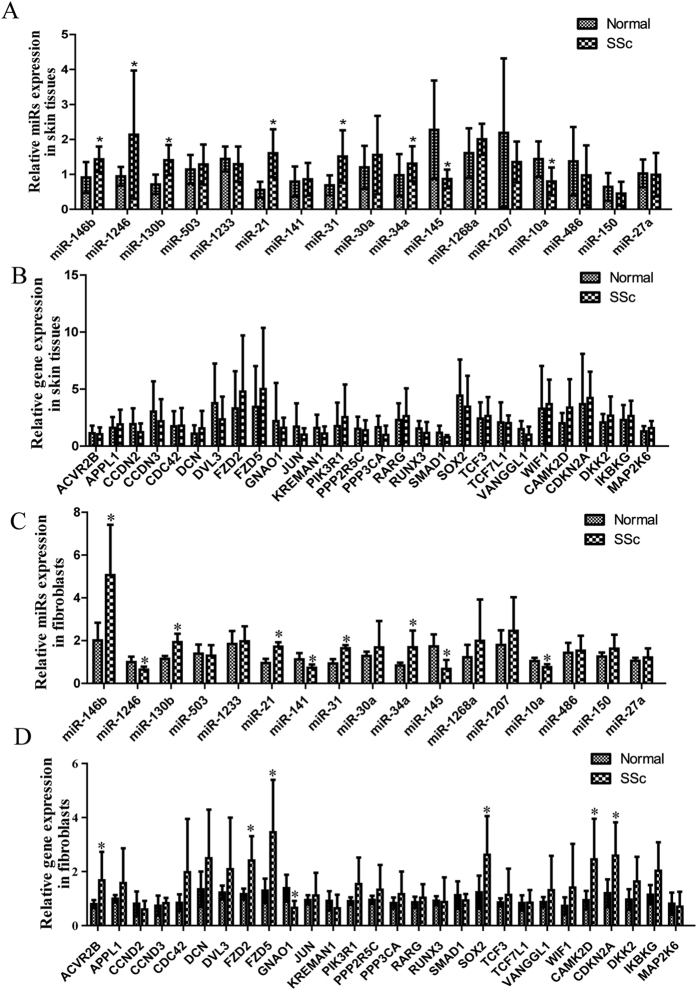
Validation of data showing differential expression of miRNAs and mRNAs. Real-time PCR was performed to analyze the levels of (**A**) miRNAs in skin tissues, (**B**) mRNAs in skin tissues, (**C**) miRNAs in skin fibroblasts, and (**D**) mRNAs in skin fibroblasts. All experiments were repeated at least 3 times. Data are presented as 2 ^(−∆ΔCT)^ relative to the levels of RNU6B or GAPDH. *Indicates p < 0.05.

To further confirm that these miRNAs and mRNAs were differentially expressed, 10 of the upregulated miRNAs, 7 of the downregulated miRNAs and their 33 target genes were selected for real-time PCR detection in SSc skin tissues and primary skin fibroblasts. In the SSc skin tissues, as demonstrated in Fig. 3A, we found that miR-146b, miR-1246, miR-130b, miR-21, miR-31 and miR-34a were expressed at substantially higher levels, while miR-145 and miR-10a were expressed at lower levels. There was no significant difference in the levels of these target genes between the two groups (Fig. 3B). In the SSc primary skin fibroblasts, miR-146b, miR-130b, miR-21, miR-31 and miR-34a were clearly expressed at higher levels, whereas miR-1246, miR-141, miR-145 and miR-10a were expressed at lower levels (Fig. 3C). The markers ACVR2B, FZD2, FZD5, SOX2, CAMK2D and CDKN2A were expressed at higher levels, whereas GNAO1 was expressed at lower levels (Fig. 3D). In summary, the expression levels of miR-146b, miR-130b, miR-21, miR-31 and miR-34a were higher while those of miR-145 and miR-10a were lower in both SSc skin tissues and primary skin fibroblasts.

### Validation of miRNAs and mRNAs that were differentially expressed in primary skin fibroblasts after stimulation with SSc serum

**Figure 4:**
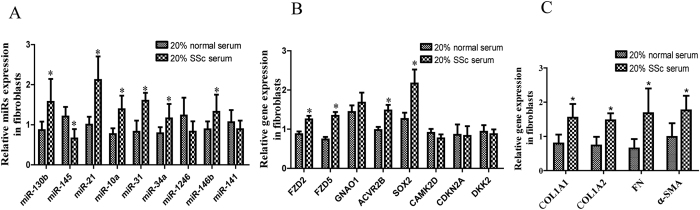
miRNAs and mRNAs were differentially expressed in fibroblasts that were stimulated with SSc serum. Fibroblasts were stimulated with 20% SSc serum for 48 h, and real-time PCR was then performed to analyze (**A**) miRNAs, (**B**) mRNAs and (**C**) fibrosis related genes. All experiments were repeated at least 3 times. Data are presented as 2^(−∆ΔCT)^ relative to the levels of RNU6B or GAPDH. *Indicates p < 0.05.

To investigate the effect of SSc serum on the activation of fibroblasts, we stimulated normal primary skin fibroblasts by applying 20% SSc serum for 48 h. We then detected the expression levels of miRNAs and mRNAs. After stimulation, the expression levels of miR-130b, miR-21, miR-10a, miR-31, miR-34a, and miR-146b were higher, while that of miR-145 was lower (Fig. 4A). The expression levels of FZD2, FZD5, ACVR2B, and SOX2 were higher (Fig. 4B). The expression of fibrosis related genes such as Collagen type I, alpha 1 and 2 (COL1A1, COL1A2), α -smooth muscle actin (α -SMA), and fibronectin (Fn) increased (Fig. 4C).

### Validation of miRNAs or mRNAs that were differentially expressed in SSc serum or human dermal microvascular endothelial cells (HMVEC) that were stimulated with SSc serum

**Figure 5:**
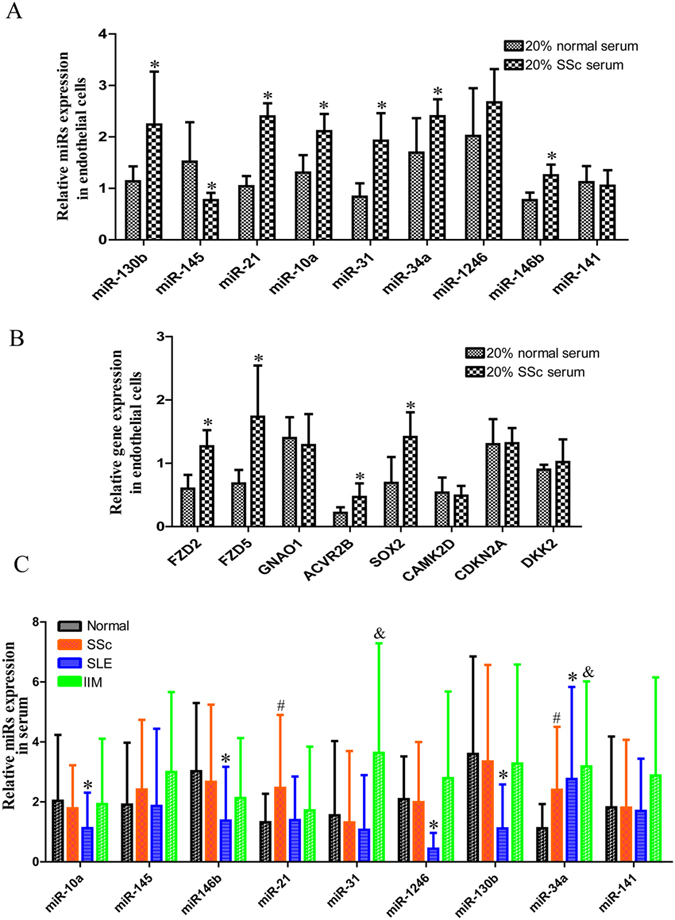
Differentially expressed miRNAs in serum and endothelial cells that were stimulated with SSc serum. Expression of (**A**) miRNAs and (**B**) mRNA in endothelial cells that were stimulated with SSc serum. (**C**) Expression of circulating miRNAs in the serum of SSc, SLE and IIM. All experiments were repeated at least 3 times. Data are presented as 2^(−∆ΔCT)^ relative to the levels of RNU6B or miR-238 or GAPDH. ^*,#,&^Indicates p < 0.05.

Extracellular miRNAs are thought to be one means of intercellular communication, and the exosome-mediated transfer of miRNA between cells has been reported in cell culture^21^. We also sought to determine the influence of SSc serum on HMVECs. After cells were stimulated with 20% SSc serum for 48 h, the expression levels of miR-130b, miR-21, miR-10a, miR-31, miR-34a, and miR-146b were higher, whereas that of miR-145 was lower in HMVECs (Fig. 5A). The expression levels of FZD2, FZD5, ACVR2B, and SOX2 were higher (Fig. 5B).

MiRNAs were detected in the circulation and can therefore serve as valuable biomarkers for the diagnosis and prognosis of various diseases in addition to providing potential therapeutic value. The expression patterns of miRNAs in the serum reflect underlying pathophysiological processes that are specific to SSc. We also detected the expression of a variety of miRNAs in the serum. MiR-21 was specifically increased in SSc serum; miR-31 was specifically increased in idiopathic inflammatory myopathy (IIM) serum; miR-10a, miR146b, miR-1246 and miR-130b were specifically decreased in systemic lupus erythematosus (SLE) serum; and miR-34a was increased in SSc, SLE, and IIM serum (Fig. 5C).

## Discussion

To our knowledge, this is the first study to integrate the effects of the dysregulation of miRNA and mRNA expression profiles in SSc. In this study, we used TargetScan to predict miRNA target mRNAs, and we combined these target mRNAs with genes that we found in other microarray data to be significantly altered. A small portion of the target genes (33/326, 10.1%) were differentially expressed in SSc skin tissues, and these were selected for further analysis. The expression levels of six of the miRNAs (miR-146b, miR-130b, miR-21, miR-31 and miR-34a) were higher, the expression level of miR-145 was lower, and the expression levels of 4 genes (ACVR2B, FZD2, FZD5 and SOX2) were identified in SSc skin tissues and fibroblasts and in normal skin fibroblasts and endothelial cells that were stimulated with SSc serum. These markers might play fundamental roles in SSc pathogenesis. However, our validation experiments did not reveal any miRNA-mRNA pairs that were negatively correlated and it’s complicated to explain. Recently, progress has been made in comparative genomics analyses and high-throughput experimental studies. The underlying miRNA-mRNA interaction networks are large, complex and only partially explored^16^. miRNAs can establish the thresholds and increase the consistency of the expression of their target genes in addition to reducing cell-to-cell variability in target gene expression^22^,^23^,^24^ and coordinating the expression patterns of various targets within individual cells^25^. However, when a miRNA’s expression is perturbed, it causes small changes in the mRNA- and protein-level expression of its predicted targets. The fraction of miRNA-induced changes in target mRNA levels is small. Few crucial targets are respond strongly to the miRNA and influence phenotypes very sensitively^16^. It should also be noted that there are many other regulation factors between the miRNAs and their target mRNAs.

Previous studies have reported that the expressions of some miRNAs, such as miR-29^13^, miR-21^12^, miR-150^26^, miR-196a^27^, let-7a^28^, miR-130b^14^ and miR- 92a^29^, are altered in SSc fibrosis. Here, we identified changes in a specific set of miRNAs, and this set of miRNAs is known to regulate the Toll-like receptor, TGF-β and Wnt signalling pathways, and they may therefore play important roles in the pathogenesis of SSc by affecting not only fibroblasts but also endothelial cells^30^,^31^. The identified circulating miRNAs (i.e., miR-21, miR-34a, miR-31, miR-10a, miR-146b, miR-1246 and miR-130b) are potentially important biomarkers for SSc, SLE and IIM. Further studies are needed to determine the roles, underlying mechanisms and potential therapeutic targets of this set of miRNAs.

The data in this study suggest that stimulation with SSc serum may activate the Wnt/β-catenin pathway in both fibroblasts and endothelial cells. A pathway analysis of miRNA targets showed that the Wnt signalling pathway was the most affected pathway. Aberrant Wnt/β-catenin signalling has been demonstrated in SSc tissues. Canonical Wnt signalling is necessary for TGF-β-mediated fibrosis because it induces the activation and differentiation of and excessive collagen release from fibroblasts^32^,^33^. The canonical Wnt/β-catenin pathway is also involved in many of the processes underlying angiogenesis, vascular remodelling and differentiation in various species and organ systems^34^. Inhibiting canonical Wnt signalling at different levels has been shown to ameliorate fibrosis in complementary mouse models of SSc. Several pharmacological inhibitors of key components of the canonical Wnt pathway have demonstrated that they induce antifibrotic effects in preclinical studies when administered in well-tolerated doses and that some of them have been associated with promising results in initial clinical trials^31^. Hence, the canonical Wnt signalling pathway might be a potential target for anti-angiogenic and/or anti-fibrotic therapies in SSc^35^,^36^.

We also found that there was no difference in the miRNA and mRNA expression profiles of SSc serum-stimulated fibroblasts and endothelial cells. We hypothesized that the components of SSc serum are complex and that they may contain circulating miRNAs enclosed in exosomes that could be transferred from the serum into the cells^37^,^38^.

In summary, these novel findings regarding miRNA and mRNA expression profiles provide hints about the mechanisms underlying SSc pathology and are therefore worth further functional analysis. The complexity of these regulatory networks is worth further study.

The limitations of this study include a lack of phenotypic information, such as modified Rodnan skin scores or data regarding the systemic involvement of SSc. In addition, we detected serum miRNAs that might act as biomarkers, but these data may not reproducible in plasma. The difference between serum and plasma miRNA concentration profiles showed that there were some associations between miRNAs obtained from platelets, potentially indicating that the coagulatory process may affect the profile of extracellular miRNAs in the blood^39^. However, both plasma and serum samples are acceptable types of specimens to analyze for circulating miRNAs. In a pilot experiment, miRNA levels were highly correlated between plasma and serum levels^40^. In our studies, we have typically worked with specimens that are processed within 2 h of collection to generate serum samples. Although miRNAs are thought to be stable over extended periods of time when incubated in plasma at room temperature (at least up to 24 h)^41^, it is not known whether this duration of time between blood collection and processing for plasma or serum affects miRNA levels^42^.

## Materials and Methods

### Ethics statement

The collection and use of human samples for this study was approved by the Ethics Committee of Central South University (No. 201303293, 201404360, 201212074), and the study was carried out according to the medical research regulations of China. Informed consent was obtained from all patients.

### Patients, biopsy specimens, and cell culture

Biopsy specimens were obtained from 14 subjects during one of our former studies, as previously described^19^. Seven patients underwent one biopsy set that consisted of a single 1-cm punch from the lateral forearm at 8 cm proximal to the ulnar malleolus. The sample was removed from clinically involved skin. Seven control samples were obtained from unaffected individuals who underwent a biopsy in the identical locations (forearm). The biopsy sample was then quartered. Two of the resulting pieces were immediately frozen, the third piece went into 10% formalin for routine histology processing, and the fourth was used for fibroblast cell culture^19^.

Primary skin fibroblasts were expanded by culturing the cells at 37 °C in a humidified atmosphere containing 95% air and 5% CO_2_ in Dulbecco’s modified Eagle’s medium (DMEM; Gibco, USA) supplemented with 10% heat-inactivated foetal bovine serum (Hangzhou Sijiqing Biological Technology, China). Cells in passages 3–5 were used in the experiments. The cells were used when they had reached 80–90% confluence.

HMVECs were obtained from normal adult skin (Cellbio, Shanghai, China) and cultured at 37 °C in an atmosphere containing 95% air and 5% CO_2_ in DMEM supplemented with 10% heat-inactivated foetal bovine serum. The cells were used when they had been cultured to 80–90% confluence.

Whole blood samples were obtained from 66 SSc patients, 37 SLE patients, 39 IIM samples and 46 normal controls. All SLE patients met the American College of Rheumatology (ACR) classification criteria for SLE^43^. IIM was diagnosed based on the criteria of Bohan and Peter^44^. Whole blood was separated into serum and cellular fractions within 2 h of the blood being drawn. Serum RNA was isolated using an miRNeasy serum kit (Qiagen) and further analyzed using real-time PCR.

### miRNA array

Data for 21 miRNAs were obtained from our previous miRNA array study^19^. As previously described, samples were sent to Beijing CapitalBio Corporation (China) for processing. The miRNAs were enriched from total RNA using an mirVana® miRNA Isolation Kit (Ambion, Foster City, CA, USA) and labelled using a FlashTag™Biotin RNA Labeling Kit. The labelled miRNAs were used to hybridize each miRNA microarray (Affymetrix). Images of the miRNA microarrays were then acquired using an Affymetrix ® Gene-Chip® Scanner 3000. The obtained signals were transformed to digital signals using image analysis software (LuxScan3.0; Capital Bio), and the free miRNA QC Tool software was used for data summarization, normalization (quantile), and quality control^19^.

### Target prediction and network analysis

The predicted target genes of the miRNAs were obtained using TargetScan v7.0. All of the target genes with conserved sites were selected. For the differentially expressed miRNAs, all of their predicted targets that were human genes and those that overlapped with differentially expressed genes were used to perform a KEGG pathway enrichment analysis. The ingenuity pathway analysis (IPA: http://www.ingenuity.com/) tool and Database for Annotation, Visualization and Integrated Discovery (DAVID) v6.7 were used to identify the networks, functions, and canonical pathways of these gene^45^,^46^. Cytoscape was used to integrate information related to the biomolecular interaction networks between these miRNAs and mRNAs.

### mRNA microarray data processing

The whole human genome oligo microarray (G4112A) data set was used to evaluate 31 SSc skin tissues and 9 normal skin tissues that were obtained from forearm skin biopsies. These data were downloaded from the Gene Expression Omnibus (http://www.ncbi.nlm.nih.gov/geo/GSE accession number GSE9285)^20^. The gene expression data was used quantile normalization, and R was used to perform a statistical test to determine whether mRNAs were differentially expressed between SSc and normal control samples. Differentially expressed mRNAs were defined those with a P-value of P < 0.05 and an at least ± 1.2-fold change between the groups.

### Treatment with SSc serum

Normal primary skin fibroblasts were cultured in serum-free DMEM (starvation medium) for 24 h and then stimulated with 20% SSc serum for 48 h. HMVECs were cultured for 24 h in serum-free DMEM (starvation medium), and then stimulated with 20% SSc serum for 48 h.

### Validation using RNA isolation and quantitative reverse transcription PCR

Total RNA was isolated from skin biopsy specimens, fibroblasts and endothelial cells using an miRNeasy mini kit (Qiagen). Serum miRNAs were isolated using a PAXgene blood miRNA system (Qiagen). An miScript reverse transcription kit (Qiagen) and miScript SYBR Green PCR kit (Qiagen) were used to measure the expression levels of selected miRNAs in a model 7500 real-time PCR system analyzer (Applied Biosystems). miRNA-specific primers and the miScript Universal Primer (Qiagen) were used. The expression of the U6B small nuclear RNA (RNU6B) was used as the endogenous control to normalize the sample data. The spiked *Caenorhabditis elegans* miRNA-238 (Qiagen, cel-miR- 238-3p) was used as an exogenous control to normalize the serum miRNA data. To detect mRNA, cDNA was prepared using a Reverse Transcription System (Promega, USA) and amplified using real-time PCR with SYBR Green (SYBR Premix Ex Taq RT-PCR kit; Takara, Shiga, Japan) using the primers shown in Supplemental Table 1. GAPDH expression was used as the endogenous control to normalize the sample data. Relative expression levels were calculated using the 2−ΔΔCt method. The miRNA primers were purchased from RiboBio (China) and are listed in Supplemental Table 3.

### Statistical analysis

GraphPad Prism software was used for all statistical analyses (not including the microarray data). Numerical variables with a normal distribution were compared using unpaired t-tests. Data with a non-normal distribution were compared using the Wilcoxon rank sum test. All data are expressed as the mean ± standard deviation (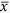 ± SD). P < 0.05 was considered to indicate statistical significance.

## Additional Information

**How to cite this article:** Zhou, B. *et al*. Integration of microRNA and mRNA expression profiles in the skin of systemic sclerosis patients. *Sci. Rep.* **7**, 42899; doi: 10.1038/srep42899 (2017).

**Publisher's note:** Springer Nature remains neutral with regard to jurisdictional claims in published maps and institutional affiliations.

## Supplementary Material

Supplementary Dataset 1

Supplementary Dataset 2

Supplementary Dataset 3
